# Survival Statistics of Digital Replantation in the UK

**DOI:** 10.7759/cureus.20183

**Published:** 2021-12-05

**Authors:** Alexander C Smith, Dariush Nikkhah, Ryckie Wade

**Affiliations:** 1 Plastic Surgery, Guy's and St Thomas' NHS Foundation Trust, London, GBR; 2 Plastic Surgery, Royal Free Hospital, London, GBR; 3 Plastic Surgery, Leeds General Infirmary, Leeds, GBR

**Keywords:** replantation, microsurgery, digital, digit, amputation

## Abstract

Background

Digital replantation is associated with a substantial risk of failure. There is considerable variation in survival rates globally, and the current data are limited by poor statistical methods and bias of selection, which limits its translation to Europe and the USA. We aimed to establish a more representative survival rate of digit replantation for western populations and evaluate espoused prognostic variables using robust statistical methodology.

Materials and Methods

Retrospective data were collected from 58 consecutive patients who underwent digital replantation following traumatic amputation in three tertiary care hand centres in the UK over seven years. The unit of analysis was the digit. Generalized linear modelling was used to estimate the odds ratio (OR) of digit survival.

Results

Forty-six of 68 replanted digits survived (68%). The typical replant candidate was a 40-year-old male manual worker. Digit survival was more likely with guillotine injuries (adjusted OR 25.5 [95% CI 5.60, 115]) and when intraoperative skeletal shortening was performed (adjusted OR 15.3 [95% CI 2.62, 89.5]). The age of the patient, seniority of the operating surgeon, and use of vein grafts was not associated with digit survival.

Conclusion

We provide robust data to show that guillotine amputations have more favourable survival rates, which can be further improved by skeletal shortening at the time of replantation. We suggest that research networks worldwide set up digit amputation registries to capture individual patient data on this uncommon injury.

## Introduction

Microsurgical techniques have revolutionised multiple facets of plastic and reconstructive surgery. Since the first successful attempt in 1968 by Komatsu and Tamai [1], digital replantation has become an option for a large proportion of the developed world. The rates of digit replantation vary considerably worldwide, from 37 per million in Taiwan [2] to 6.6 per million in the UK [3] and 4.3 per million in the USA [4]. Furthermore, cultural and occupational differences between persons in different countries introduce considerable selection bias, which is likely to reduce the generalisability of studies between countries.

The landscape continues to change with regard to our understanding of digital replantation. As techniques and success rates improve [5-7], the previously held contraindications to replantation are now being challenged [8]. Large studies from China are now reporting success rates between 84%-96% [9] whereas, in comparison, recent UK data presents a success rate of 68%-70% [3,10]. These papers also attempt to identify predictive factors for replanting survival, but these papers lack adequate statistical analysis, fail to appropriately model-related data from individuals with multidigit replantation, and present comparatively small samples from single centres.

Therefore, we aim to evaluate the outcomes of digit replantations in three tertiary care hand trauma centres in the United Kingdom and establish the factors that affect replant survival.

## Materials and methods

This retrospective cohort study includes consecutive patients who underwent digital replantation following traumatic amputation in three tertiary care hand centres in the UK between May 2010 and May 2017. 

We included patients of all ages with single or multiple digit amputations for whom replantation was attempted. Those who underwent microsurgical vascular repair met the inclusion criteria. We did not include composite grafts or any injury that maintained a bridge of tissue. The decision to undergo replantation in each of the three centres was a joint decision with the surgeon and patient, taking into account the relative indications and contraindications recognised in the literature [11]. Replantation was not attempted on all amputated digits.

Replantation success was defined as tissue survival. Replantation failure was defined as auto or operative terminalisation, whether due to medical indication or patient choice within the minimum one-month follow-up period. Replantation level was recorded using the Tamai classification system [12]. Regarding the mechanism of injury, cases were placed into one of three categories: guillotine, crush, and avulsion. ‘Ischaemia time’ denotes the period between the time of injury and the start of the operation, as the time of the microsurgical anastomosis is rarely recorded but can be reasonably estimated from the procedure start time. A procedure with an operating start time between 5 pm and 8 am was categorised as ‘out-of-hours’.

For statistical analysis, the unit of analysis was the digit. This study does not seek to test a hypothesis but rather to capture the prevalence of outcomes in normal service, so there is no role for a sample size calculation. Continuous data approximating the normal are presented as means with standard deviations (SD). Skewed and count data are summarised by the median and interquartile range (IQR). Mixed-effects regression was used to estimate the associations between replant survival and predictors. The binomial family was used. The covariances were distinctly estimated (unstructured). The fixed-effects for the multivariable model were selected priorly (based upon risk factors identified in the literature [13]) as age in years, the mechanism of injury, grade of an operating surgeon operating, bone shortening, the use of vein graft(s) and perioperative anticoagulation or antiplatelets. The strata (random-effect) was the patient. Cluster robust standard errors (and 95% confidence intervals, CI) were generated. 

Permission to collect this data was granted at each of the involved institutions. Neither patient consent nor ethical review was required for this service evaluation following the Health Research Authority's guidance.

## Results

In 58 adults, 46 of 68 replanted digits survived replantation (68%). Table 1 summarises differences in baseline characteristics. The typical replant candidate was a 40-year-old male manual worker. Patients sustaining a guillotine-type injury were a mean 17 years older (95% CI 3, 31) than those suffering crush or avulsion injury. Relevant peri-operative steps during digit replantation are shown in Table 2. 

**Table 1:** Baseline characteristics

		Replanted Digits		
		Failure (n=22)	Survived (n=46)	p-value
Mean age in years (SD)		32 (21)	44 (19)	0.020
Sex (%)	Male	19 (86)	41 (89)	0.742
	Female	3 (14)	5 (11)	
Current Smoker (%)		5 (23)	12 (29)	0.583
Manual worker (%)		11 (52)	30 (68)	0.217
Amputated digit	Thumb	5 (23)	17 (37)	0.087
	Index	2 (9)	13 (28)	
	Middle	9 (41)	9 (20)	
	Ring	4 (18)	6 (13)	
	Little	2 (9)	1 (2)	
Mechanism of injury (%)	Guillotine	5 (23)	31 (67)	0.003
	Avulsion	4 (18)	4 (9)	
	Crush	13 (59)	11 (24)	
Tamai level (%)	1	1 (5)	3 (7)	0.821
	2	7 (32)	11 (24)	
	3	7 (32)	10 (22)	
	4	7 (32)	16 (35)	
	5	0 (0)	6 (13)	

**Table 2:** Peri-operative events

Factor	Digits Replanted		
	Failure (n=22)	Survived (n=46)	p-value
Consultant operating (%)	11 (50)	19 (42)	0.535
Vein graft used (%)	7 (32)	11 (24)	0.500
Bone shortening (%)	6 (27)	25 (54)	0.042
Mean* ischaemia time in minutes (95% CI)	380 (331, 437)	365 (277, 481)	0.787
Mean operative time in minutes (SD)	398 (167)	422 (199)	0.591
Postoperative leech therapy (%)	8 (38)	7 (15)	0.056
Perioperative anticoagulation (%)	10 (91)	27 (87)	0.752
Postoperative antiplatelet (%)	1 (5)	2 (4)	0.974
Median inpatient stay in days (IQR)	4.5 (4-7)	7 (2.5-10)	0.095

**Figure 1:**
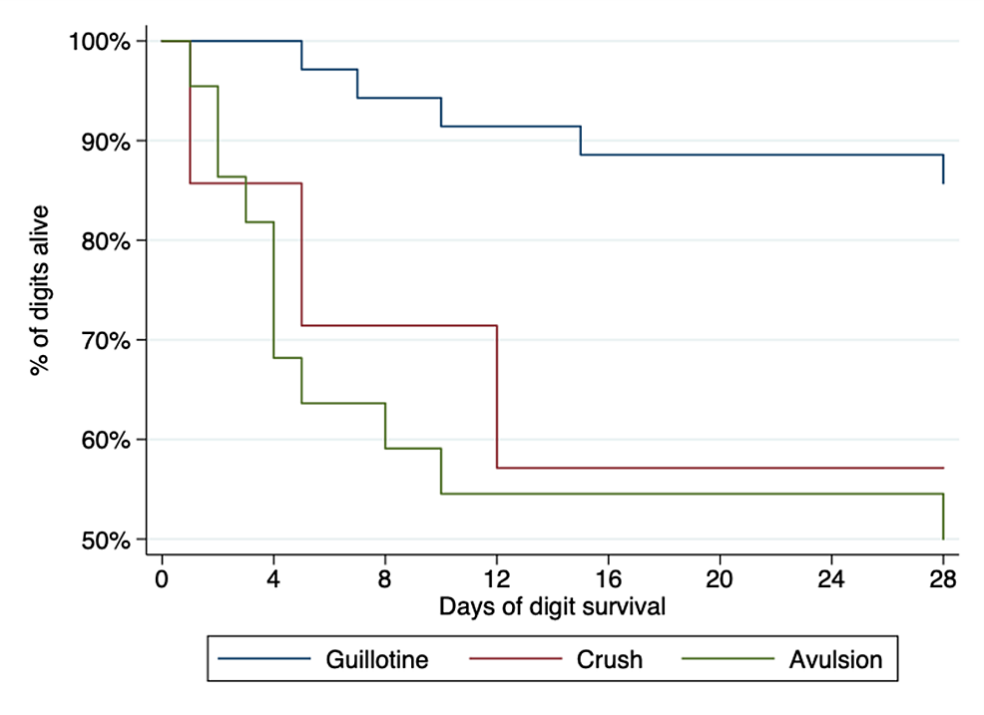
Kaplan-Meier plot showing that replanted digits which were traumatically amputated by a guillotine-type mechanism have the best survival profile, as compared to those amputated by crushing or avulsion injuries

**Figure 2:**
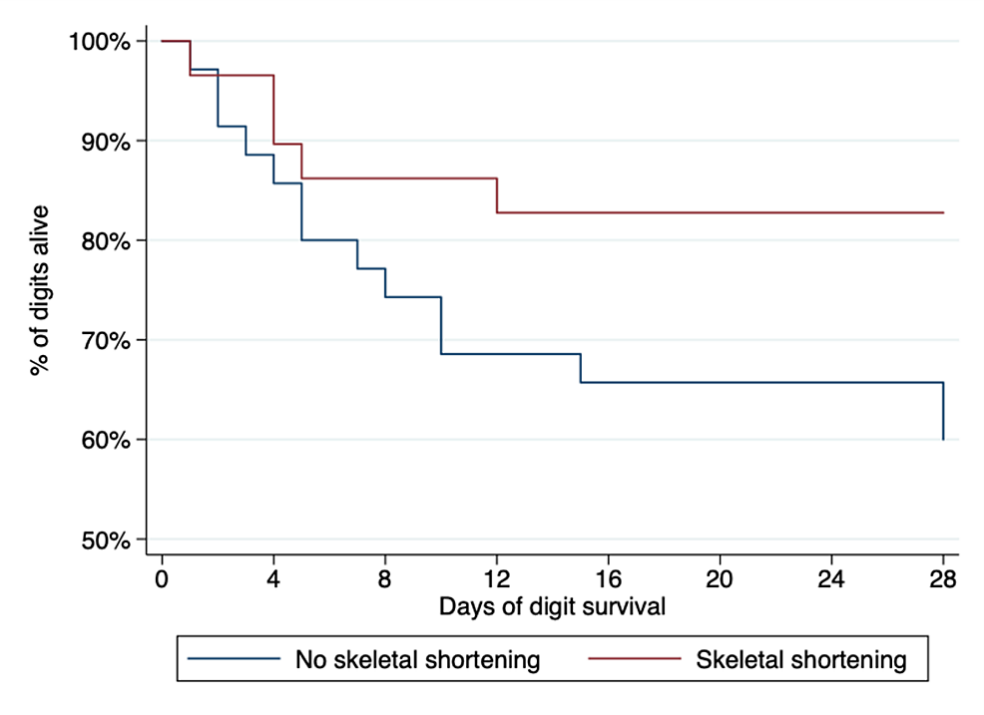
Kaplan-Meier plot showing that skeletal shortening at the time of replantation improves the probability of survival

**Figure 3:**
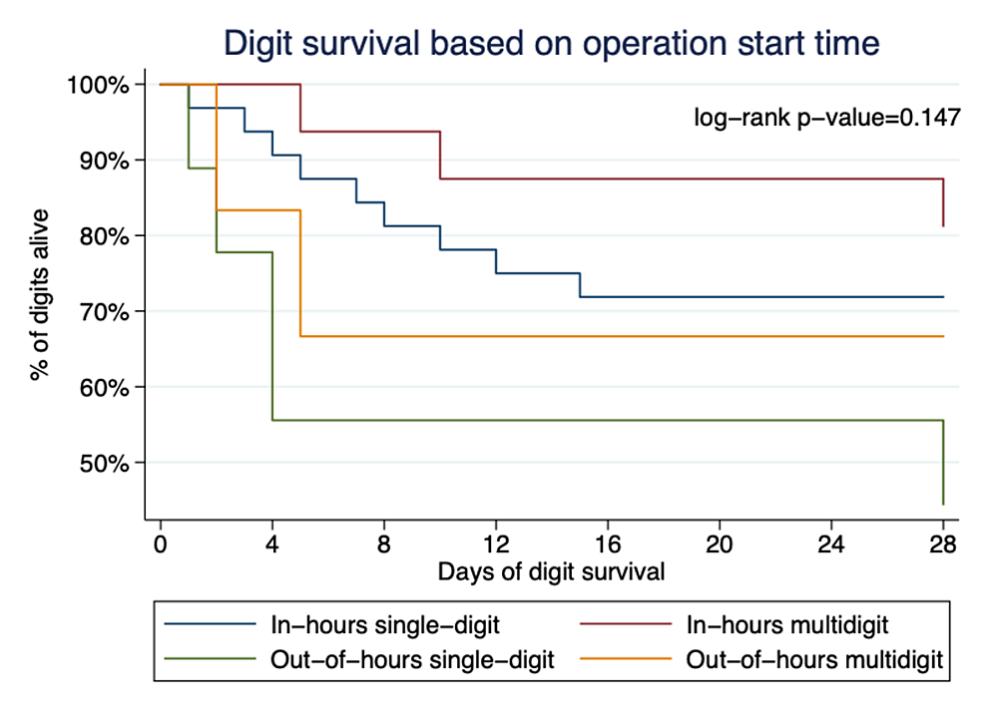
Kaplan-Meier plot showing that the survival of single and multi-digit replants according to the start time of surgery

Our mixed-effects model shows that the survival of a replanted digit was strongly related to the mechanism of injury and intraoperative skeletal shortening (Table 3). After adjustment for other factors, amputation by a clean-cut (guillotine) mechanism was associated with 25 times the odds of success compared to replanting avulsed or crushed digits (Figure 1). The use of skeletal shortening improved the odds of digit survival by 15-fold (Figure 2). Despite the apparent univariable association between operative start time and digit survival (Figure 3), there was no statistically significant difference between groups in the mixed-effects model. Approximately 2% of the variation (95% CI 1.17%, 3.53%) in digit survival was due to the number of amputated digits (within patient factors), meaning that the survival of multidigit replantation appears to be very similar to single digits.

**Table 3:** Mixed effects logistic regression for the odds of digit survival

Perioperative factors		Odds of Replanted Digit Surviving			
		Unadjusted odds ratio (95% CI)	p-value	Adjusted odds ratio (95% CI)	p-value
Age in years		1.03 (1.01, 1.06)	0.020	0.99 (0.98, 1.06)	0.257
Guillotine mechanism		7.0 (2.3, 21)	0.001	25.5 (5.60, 115)	<0.001
Tamai level*	1	0.52 (0.04, 6.23)	0.822	23.7 (1.8, 302)	0.048
	2	0.48 (0.04, 5.41)		22 (1.9, 259)	
	3	0.76 (0.07, 8.41)		4.3 (0.58, 32.7)	
	4	referent		referent	
Consultant Operating		0.73 (0.27, 1.97)	0.535	0.40 (0.08, 1.92)	0.251
Vein Graft used		0.67 (0.21, 2.12)	0.500	0.80 (0.12, 5.25)	0.812
Bone shortening		3.17 (1.04, 9.78)	0.042	15.3 (2.62, 89.5)	<0.002
Ischaemia Time		0.99 (0.99, 1.00)	0.787	1.00 (0.99, 1.00)	0.783
Out of hours operating		0.43 (0.14, 1.32)	0.143	0.86 (0.12, 5.96)	0.877

Of the 22 replanted digits which did not survive perioperatively, 11 (65%) were due to arterial insufficiency, five (29%) were due to venous congestion, and the reasons were mixed in the remaining five (29%). After 46 days, one patient was elected to undergo revision terminalisation due to slow healing, cold intolerance, and pain.

At a median of seven months, follow-up, the replanted 46 digits belonging to 44 patients had the following outcomes: nine (20%) were cold intolerant, four (9%) were hypersensitive, six (13%) had paraesthesia in one of both digital nerve territories, two (4%) were painful, and eight (17%) had a stiffness of the interphalangeal joint(s). 

## Discussion

We have shown that approximately one in three replanted digits ultimately failed. We provide evidence that replantation survival was more favourable for distal amputations sustained by a guillotine mechanism (clean cut) and when skeletal shortening was performed at the time of replantation.

Our findings concur with recent studies from the United Kingdom, which reported survival rates of 68%-70% [3,10]. However, it is noted that replantations in the UK demonstrated less favourable outcomes when compared to results abroad [9,13]. The reasoning for this discrepancy is likely multifactorial but may be related to differing mechanisms of injury and different patient priorities in the decision-making process. Equally, the incidence is likely to be lower in the UK given the health and safety standards, equipment, regulation, and legislation involving machinery in manufacturing [14]. The potential for selection bias (surgeons choosing not to report failures), publication bias (journals favouring studies with higher survival rates), and patient selection are other factors that can only be explored through evidence synthesis. Although, regarding the latter, due in part to cultural factors [15-17], surgeons in the Far East are more likely to attempt a digital replantation despite potentially unfavourable pre-operative factors. One might assume that this approach would lead to a lower replantation success rate; however, the evidence does not support this assumption [18].

The mechanism of injury had a significant impact on the survival of the replanted digit. Amputation by a clean-cut (guillotine) mechanism was associated with 25 times the odds of survival compared to replanting avulsed or crushed digits (Figure 1). A large metanalysis of digital replantation from 2018 identified the mechanism of injury as one of the most significant factors for replanting survival [18]. Choi et al. [19] described how significant crush injury should be a contraindication to replantation; however, our study is not alone in showing that many patients with digital amputation after crush injury continue to put forward as surgical candidates [9]. We suggest the condition of the remaining tissues adjacent to the zone of injury should be one of the first factors to consider when discussing replantation with the patient.

Several other perioperative factors were assessed (Table 2) but were not statistically associated with digit survival. Initially, the data suggested that increasing age was favourable, but this was confounded by the mechanism of injury, whereby older persons were more frequently subject to guillotine injuries. This again highlights the need to perform multivariable modelling to understand the true effects of several co-variables on the outcome of interest.

Intra-operatively, bone shortening is widely accepted as a method of reducing tension on the soft tissues and anastomoses [11]. Our results corroborated this view, and we provided evidence that skeletal shortening increased the odds of survival 15-fold (Figure 2). The confidence interval is wide, which means the benefit could be as low as twice as good or up to 90 times better; this is likely to be due to the case-mix, whereby shortening Tamai 1/2 guillotine injuries is likely to provide minimal benefit whereas shortening more proximal and non-clean-cut amputations may provide substantial benefit. Whilst we did not collect information on the amount of bone shortening (i.e., we are unable to differentiate 1mm from 10mm shortening), which is important for clinical translation, we provide robust evidence of benefit from bone shortening in all circumstances. Future researchers should consider measuring the amount of shortening performed such that future articles can provide evidence-based answers to the question of “how much is enough?”. We also observed that arterial repair with vein grafting did not affect the odds of digit survival in either the univariable or adjusted models, meaning that in this dataset, it does not appear to affect survival, in line with recent literature [20].

One important weakness of this study is that it focuses on tissue survival and not function, which is arguably the more relevant outcome measure. The reason for this was twofold. Firstly, tissue survivability is the natural starting point for this topic, and given the variability in the literature, we chose this as the primary outcome. It is reasonable to suggest that a study focussing on functional outcomes without assessing and addressing tissue survival would be premature. Secondly, on evaluation of the documentation, it became clear that patient notes were often missing patient-reported outcome data or were not recorded in the first place, and this made any evaluation of functional outcomes for this uncommon injury difficult. 

Ultimately, the replant success rates demonstrated in this study fall short of series seen in other centres abroad, and this raises several important questions. There are a vast number of intra-operative and peri-operative factors that may influence the survival of a digital replant, and we have shown that mechanisms of injury and bone shortening are significant. But given the scarcity of cases [21], the hand surgery community should focus on pooling data where possible to identify other significant factors that not only affect the survivability of a digit but the functionality too.

To support the global push for open science, which in turn facilitates individual patient data meta-analysis (which is particularly important for uncommon entities like digit replantation), the authors have made the anonymised raw data freely available via the Open Science Framework [22]. The statistical syntax can be obtained from the third author. Furthermore, we propose the concept of a digital amputation registry via ever-growing collaborative research communities. It will be designed to collect robust functional and patient-reported [23] outcome data from UK hand centres to facilitate the replant vs terminalisation discussions for future surgeons and patients.

## Conclusions

We provide robust data to show that guillotine amputations have more favourable survival rates, which can be further improved by skeletal shortening at the time of replantation. We suggest that research networks worldwide set up digit amputation registries to capture individual patient data on this uncommon injury.
